# Caloric Intake with High Ratio of Enteral Nutrition Associated with Lower Hospital Mortality for Patients with Acute Respiratory Distress Syndrome Using Prone Position Therapy

**DOI:** 10.3390/nu13093259

**Published:** 2021-09-18

**Authors:** Pin-Kuei Fu, Wen-Cheng Chao, Chiann-Yi Hsu, Chih-Hung Wang, Chen-Yu Wang

**Affiliations:** 1 Department of Critical Care Medicine, Taichung Veterans General Hospital, Taichung 40705, Taiwan; yetquen@gmail.com (P.-K.F.); cwc081@hotmail.com (W.-C.C.); 2 Ph.D. Program in Translational Medicine, National Chung Hsing University, Taichung 402010, Taiwan; 3 College of Human Science and Social Innovation, Hungkuang University, Taichung 43302, Taiwan; 4 Department of Computer Science, Tunghai University, Taichung 407224, Taiwan; 5 Department of Automatic Control Engineering, Feng Chia University, Taichung 40780, Taiwan; 6 Biostatistics Task Force of Taichung Veterans General Hospital, Taichung 40705, Taiwan; chiann@vghtc.gov.tw; 7 Graduate Institute of Education, National Changhua University of Education, Changhua 50007, Taiwan; chiwang@cc.ncue.edu.tw; 8 Department of Nursing, Hungkuang University, Taichung 43302, Taiwan

**Keywords:** acute respiratory distress syndrome, prone position, mortality, critically ill patients, enteral nutrition

## Abstract

Positioning patients in the prone position leads to reduced hospital mortality rates for those with severe acute respiratory distress syndrome (ARDS). What constitutes the optimal feeding strategy for prone patients with ARDS is controversial. We conducted a retrospective study that enrolled 110 prone patients with ARDS in two medical intensive care units (ICUs) from September 2015 to November 2018. Inclusion criteria were as follows: age ≥20 years, diagnosis of respiratory failure requiring mechanical ventilation, diagnosis of ARDS within 72 h of ICU admission, placement in a prone position within the first 7 days of ICU admission, and ICU stay of more than 7 days. Exclusion criteria were as follows: nil per os orders because of gastrointestinal bleeding or hemodynamic instability, and ventilator dependency because of chronic respiratory failure. The consecutive daily enteral nutrition(EN)/EN + parenteral nutrition(PN) ratio could predict hospital mortality rates within the first 7 days of admission when using generalized estimating equations (*p* = 0.013). A higher average EN/EN + PN ratio within the first 7 days predicted (hazard ratio: 0.97, confidence interval: 0.96–0.99) lower hospital mortality rates. To reduce hospital mortality rates, caloric intake with a higher EN ratio may be considered for patients in prone positions with ARDS.

## 1. Introduction

Acute respiratory distress syndrome (ARDS) was first described in 1974 [1], with hospital mortality rates of approximately 30–40%, depending on the severity of ARDS [2]. Common risk factors of ARDS are pneumonia, non-pulmonary sepsis, aspiration, non-cardiogenic shock, pancreatitis, severe trauma, drug overdose, and ischemic perfusion injury [3]. ARDS patients in acute stage are at risk of barotrauma, nosocomial infection, muscle weakness, gastrointestinal bleeding, delirium, and poor nutrition [4,5,6,7,8,9]. The survivors of ARDS also experience both physical and psychological impairments [4,10]. Guiilen et al. conducted a randomized controlled study that concluded that the prone position can decrease hospital mortality rates for patients with severe ARDS [11].

Enteral nutrition (EN) is the preferred method for delivering nutrients to patients in the intensive care unit (ICU) [12]. EN in critical illness is associated with many advantages including reduced inflammation, regain of muscle function, provision of micro- and macronutrients, maintenance of gut integrity, and promotion of insulin sensitivity [13]. To achieve the optimal energy goal, parenteral nutrition (PN) might be delivered during an ICU stay, but PN is not recommended during the early stages of critical illnesses [14]. It is already known that conservative fluid management can decrease the number of days a patient depends on a ventilator, whereas full caloric feeding is typically not more beneficial than trophic feeding for patients with ARDS [9,15]. ARDS patients in a prone position are still at a higher risk of posture-related feeding intolerance (FI) [16,17]. By prescribing PN to achieve energy requirements, clinicians take the risk of unnecessary fluid infusion and calories through venous routes.

What constitutes the optimal feeding strategy for patients in prone positions with ARDS remains controversial. Whether excess PN has harmful effects is unknown. As reported herein, we conducted a retrospective study to explore the energy route of ARDS patients in prone positions. We hypothesized that EN, not PN, is the preferred route of energy delivery for patients in prone positions with ARDS.

## 2. Materials and Methods

### 2.1. Study Design and Patients

This was a retrospective study conducted at two medical ICUs in a tertiary medical center in central Taiwan. The study was approved by the Institutional Review Board of Taichung Veterans General Hospital (IRB No. CE19327A). Informed consent was waived because the data were collected retrospectively from medical charts. Inclusion criteria for patients were as follows: age ≥20 years, diagnosis of respiratory failure requiring mechanical ventilation, diagnosis of ARDS within 72 h after ICU admission, placement in a prone position within the first 7 days of ICU admission, and ICU stay of more than 7 days. Exclusion criteria were as follows: nil per os orders because of gastrointestinal bleeding or hemodynamic instability, and ventilator dependency because of chronic respiratory failure. Eligible participants were enrolled from September 2015 to November 2018.

ARDS diagnosis criteria included newly onset development within 1 week, ability to explain bilateral opacity by pleural effusion, collapse, or lung nodules, and exclusion of cardiogenic hydrostatic edema by heart echography. Severity was separated into mild, moderate, and severe according to PaO_2_:FiO_2_ ratio.

Patients were placed in a prone position when their ratio of partial pressure of oxygen in arterial blood (PaO_2_) to fractional inspired oxygen concentration (FiO_2_) was less than 150 mmHg and their FiO_2_ was at least 0.6. The type of prone position was a continuous prone position [18]. The feeding procedure comprised continuous pump feeding and prophylactic prokinetic agent usage. The gastric residual volume was 250 cc, and the initial target caloric goal was 25 kcal/kg/day. Clinical physicians made decisions regarding the timing of and decision to add PN.

### 2.2. Data Collection and Outcomes

Basic demographic data including age, sex, comorbidity, daily energy intake from days 1 to 7 (both EN and PN), modified Nutrition Risk in the Critically Ill (mNUTRIC) scores [19], Acute Physiology and Chronic Health Evaluation II (Apache II) scores [20], and Sequential Organ Failure Assessment scores [21] were recorded. Outcome measurements included the hospital mortality rate and the number of days of ICU stay, hospital stay, and ventilator dependency.

### 2.3. Statistical Analysis

The data were processed using SPSS (version 22.0; International Business Machines Corp, Armonk, NY, USA). Categorical variables were presented as numbers and percentages, and differences were expressed using the chi-square and Fisher’s exact tests. Continuous variables were presented as means and SDs, and differences were expressed using the Mann–Whitney U and Kruskal–Wallis tests. Multivariate logistic regression was performed to determine the odds ratio and 95% confidence intervals for hospital mortality. Generalized estimating equations were used to estimate the association between hospital mortality rates and the daily EN/PN + EN ratios. All tests were performed using two-sided tests, and a *p*-value <0.05 was considered statistically significant.

## 3. Results

### 3.1. Patients’ Demographic Characteristics

**Figure 1:**
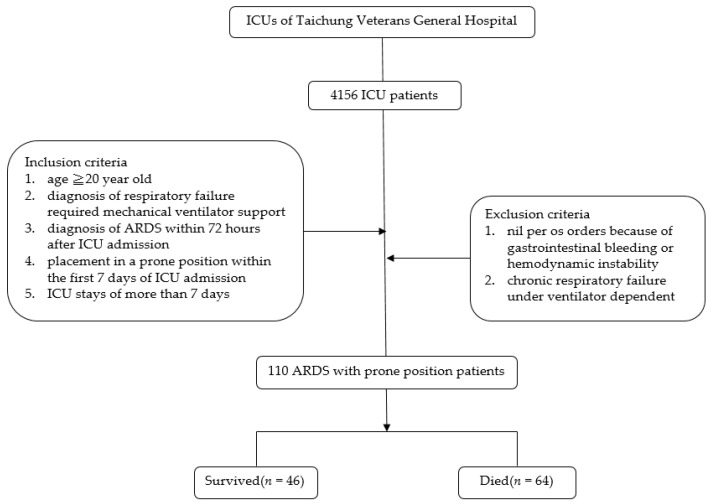
Flow chart of patients in the study. ICU: intensive care unit; ARDS: acute respiratory distress syndrome.

We screened 4156 ICU patients, and 110 patients enrolled in the study (Figure 1). Table 1 lists patients’ basic demographic data, caloric intake, and comorbidities. The overall hospital mortality rate was 58.2%. The lowest average 7 day EN intake and highest average 7 day PN intake were associated with high mortality rates, but the average 7 day total caloric intake was not associated with a high mortality rate. However, the average 7 day EN/EN + PN ratio was 84.7% in patients who survived and 69.8% in patients who died (*p* < 0.001). Advanced age, high Apache II score, low body weight, and high mNUTRIC score were associated with higher mortality rates. The most common comorbidity was hypertension (Table 1).

### 3.2. Daily EN, PN, and EN/EN + PN Ratios and Hospital Mortality

**Figure 2:**
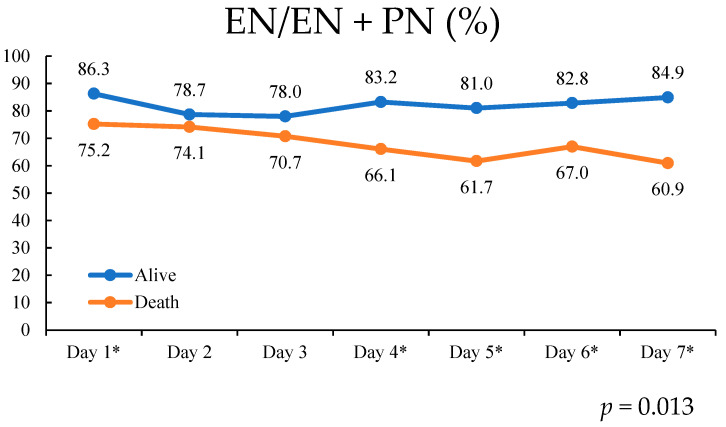
Daily EN/EN + PN ratios in the first 7 days after admission; comparison of survival with mortality by generalized estimating equations (*p* = 0.013). Higher EN/EN + PN ratios on day1, 4, 5, 6, and 7 were associated with lower hospital mortality rate (* *p* < 0.05).

Table 2 lists the first 7 day average daily caloric intake for patients who both survived and died. The higher daily EN intakes on days 4, 5, and 7 were associated with lower hospital mortality rates. By contrast, higher daily PN intakes on days 1, 4, 5, 6, and 7 were associated with higher hospital mortality rates. We calculated the daily EN/EN + PN ratio and determined that higher daily EN/EN + PN ratios on days 1, 4, 5, 6, and 7 were associated with lower hospital mortality rates (Table 2, Figure 2).

The consecutive daily EN/EN + PN ratio predicted hospital mortality rates within the first 7 days of admission when using a generalized estimating equation (*p* = 0.013; Figure 2).

### 3.3. Adjusted Hazard Ratios of Hospital Mortality

In the multivariate logistic regression model, the Apache II score and EN/EN + PN ratio were predictors of hospital mortality rates. A higher average EN/EN + PN ratio in the first 7 days was a predictor (HR: 0.97, CI: 0.96–0.99) of lower hospital mortality after age, Apache II scores, and sex were adjusted (Table 3).

## 4. Discussion

EN is the preferred method of caloric delivery for critically ill patients [12,13]. Our results indicated that a higher EN ratio is associated with lower hospital mortality rates for patients in prone positions with ARDS. However, PN is more easily applied for critically ill patients than EN [22]. FI is one reason to prescribe PN for critically ill patients. It is defined by a large gastric residual volume, presence of gastrointestinal symptoms, or inadequate delivery of EN [23]. The prevalence of feeding intolerance is approximately 22–57% in critically ill patients. FI is associated with higher mortality rates, nosocomial infection, and longer ICU stays [23]. The prevalence of FI in patients in prone positions with ARDS is unknown but might be higher compared with other critically ill patients. To facilitate EN usage in critically ill patients, feeding intolerances should be overcome as soon as possible. Implementing feeding protocols is a practical strategy to optimize EN efficiency [24,25]; it improved the estimated caloric delivery from 57.7% to 70.3% in our previous study [25]. However, the actual caloric intake in our present study was lower compared with that of our previous study. One possible explanation is our failure to implement post-pyloric access for most patients with poor intake. In our institution, gastroenterologists usually hesitate to insert post-pyloric tubes in patients in prone positions who have high oxygen needs. Otherwise, post-pyloric access has increased EN intake quantities [12,26].

Several disadvantages of early PN exist for patients in the ICU. Early PN within 7 days of ICU admission is associated with higher incidence of both infections acquired in the ICU and invasive fungal infections [14,27]. PN is supposed to deliver more glucose and calories [28]. Blocking inner glucose utilization and suppressing autophagy are two methods that result in poor outcomes in the early stages of critical illness [29,30,31]. Our results are consistent with the disadvantages of PN. A higher percentage of EN as an energy source was associated with a lower hospital mortality rate. Significant differences were observed on days 1, 4, 5, 6, and 7. Although the “Impact of Early Enteral vs. Parenteral Nutrition on Mortality in Patients Requiring Mechanical Ventilation and Catecholamines” study identified more gastrointestinal complications from EN among patients in the early stages of septic shock [32], the caloric intake of the study’s participants was close to that of full caloric feeding. According to the recommendation of the European Society for Clinical Nutrition and Metabolism guidelines, less than 70% of energy intake in the acute phase of critical illness is optimal [26]. The caloric intake revealed in our results was approximately 46% of the estimated caloric intake of the groups of patients who died and survived. Prone position-related feeding intolerance might explain the lower level of caloric intake. Nevertheless, the “Permissive Underfeeding or Standard Enteral Feeding in Critically Ill Adults” study revealed that permissive underfeeding was not inferior to full caloric feeding in patients with 90 day mortality [33]. The actual caloric intake for the permissive feeding group was approximately 50% of predicted energy. The total caloric intake in our results was very close.

Few studies have discussed the relationship between energy intake and clinical outcomes for patients with ARDS. A retrospective analysis showed that a higher energy intake within the first 6 days of ICU admission was associated with a higher mortality rate [34]. Another retrospective study revealed that a higher degree of organ failure coupled with higher caloric delivery is associated with higher mortality rates among patients with ARDS [35]. A daily caloric intake of less than 11.5 kcal/kg/day is associated with a lower hospital mortality rate than a high caloric intake. The actual caloric intake in our study was 46% of the estimated caloric intake of approximately 11.5 kcal/kg/day. These results are compatible with the “less is more” concept of caloric delivery in patients who are critically ill [36]. Our data suggest that a high EN ratio is further associated with lower hospital mortality rate for patients in prone positions with ARDS.

The prone position is associated with lower mortality rates for patients with ARDS [11] and has been an emphasized technique in the COVID-19 pandemic era [37,38]. Although ESPEN guidelines and studies consider feeding intolerance common in patients in prone positions [26], feeding patients in prone positions with ARDS is challenging. However, most studies were conducted using small case numbers [39]. A prospective study that enrolled 34 patients demonstrated that feeding ARDS patients in the prone position did not significantly increase gastrointestinal complications [40], and the feeding amount could reach 92% of the estimated caloric intake. The mean age of that study’s participants was 47.6 years, much younger than that of our patients. The Apache II score in the study was also lower than ours. Older age and higher disease severity might account for the caloric intake differences in our results. Delayed gastric emptying-related high residual gastric volume and more vomiting episodes were observed from a critical nursing care’s viewpoint [41]. Our study encompassed more cases than previous studies did and echoed the suboptimal caloric delivery. Because of the prone position and high severity of ARDS, a higher frequency of feeding intolerance in ARDS patients in prone positions than in other patients is predictable.

For patients with feeding intolerance, both ASPEN and ESPEN guidelines recommend starting EN early and feeding patients through post-pyloric tubes [12,26]. Randomized controlled trials that include large case numbers must be conducted to determine whether post-pyloric tubes should be considered first to increase feeding efficiency, as well as establish whether the caloric goal can be reached by using a specific feeding protocol, including earlier post-pyloric tube feeding for patients in prone positions that might facilitate EN rather than additional PN.

To our knowledge, our study has evaluated the most cases of patients in prone positions with ARDS regarding EN. However, our study inevitably had some limitations. Firstly, this was a retrospective study and, thus, not all variables could be controlled. The present results, suggesting a causal relationship between the EN ratio and mortality rate in ARDS patients in prone positions, must be verified through further larger clinical trials. Secondly, we did not report the amount of protein intake because it could not be acquired from the medical records retrospectively. Thirdly, the feeding protocol could not be executed completely because inserting post-pyloric tubes in patients in prone positions is difficult. Fourthly, because we enrolled patients from medical ICUs only, the results would be difficult to apply to surgical or mixed ICUs. Lastly, we did not use indirect calorimetry to calculate precise caloric consumption.

Nonetheless, our study had several strengths. Firstly, patients’ caloric intake in the study was close to 50% of the estimated caloric intake, which, according to the literature, is an acceptable caloric intake alternative to full caloric intake. Secondly, our study is the first to emphasize the EN ratio in patients in prone positions with ARDS. Lastly, regarding calorie delivery, we evaluated more cases of ARDS patients in prone positions than previous studies did.

## 5. Conclusions

EN is the preferred method of caloric delivery to ICU patients. Our present results echoed the advantages of EN in critically ill patients. To reduce hospital mortality rates, optimizing caloric intake with an increased EN ratio may be considered for patients in prone positions with ARDS.

## Figures and Tables

**Table 1:** Demography of patients in prone position with ARDS.

	Alive (*n* = 46)	Mortality (*n* = 64)	*p*-Value
	Mean ± SD	Mean ± SD	
Age (year)	55.6 ± 18.9	65.3 ± 14.2	0.003 **
Apache II	28.9 ± 6.3	32.3 ± 6.6	0.006 **
**Sex (*n*, %)**			0.362
Male	30 (65.2%)	35 (54.7%)	
Female	16 (34.8%)	29 (45.3%)	
Weight (kg)	69.2 ± 16.3	61.4 ± 12.7	0.003 **
Body mass index (kg/m^2^)	25.4 ± 5.4	23.9 ± 4.2	0.060
Average 7 day EN (kcal/day)	671.7 ± 281.5	546.6 ± 340.8	0.033 *
Average 7 day PN (kcal/day)	102.9 ± 86.8	179.0 ± 129.7	0.001 **
Average 7 day EN + PN (kcal/day)	774.7 ± 269.6	725.6 ± 322.8	0.449
Average 7 day EN/EN + PN (%)	84.7 ± 14.6	69.8 ± 23.1	<0.001 **
SOFA	11.2 ± 3.6	11.5 ± 3.3	0.491
Days of hospital stay (days)	37.8 ± 23.8	23.7 ± 18.8	<0.001 **
Days of ICU stay (days)	21.1 ± 11.2	15.1 ± 9.8	0.005 **
Days of ventilator dependency (days)	22.9 ± 16.1	16.9 ± 10.8	0.061
**Comorbidity (*n*, %)**			
Diabetes mellitus	15 (32.6%)	20 (31.3%)	1.000
Hypertension	20 (43.5%)	25 (39.1%)	0.789
Congestive heart failure ^f^	3 (6.5%)	5 (7.8%)	1.000
Liver cirrhosis	5 (10.9%)	9 (14.1%)	0.837
COPD	7 (15.2%)	8 (12.5%)	0.898
Immunocompromised host	8 (17.4%)	18 (28.1%)	0.280
Hemodialysis	18 (39.1%)	23 (35.9%)	0.887
Sepsis (*n*, %)	23 (50.0%)	40 (62.5%)	0.266
mNUTRIC Score	5.6 ± 1.6	6.6 ± 1.5	<0.001 **
High nutritional risk (*n*, %)	36 (78.3%)	60 (93.8%)	0.034 *

Values are means ± standard deviation; APACHE II: Acute Physiology and Chronic Health Evaluation II; EN: enteral nutrition; PN: parenteral nutrition; SOFA: Sequential Organ Failure Assessment; ICU: intensive care unit; COPD: chronic obstructive pulmonary disease; * *p* < 0.05, ** *p* < 0.01.

**Table 2:** Daily EN, PN, and EN/EN + PN ratios and hospital mortality.

	Alive	Mortality	*p*-Value
EN (kcal/day)			
Day1	431.1	447.7	0.798
Day2	512.5	494.8	0.598
Day3	606.0	556.6	0.436
Day4	732.1	590.8	0.028 *
Day5	756.5	544.8	0.003 **
Day6	800.9	644.4	0.090
Day7	845.7	546.9	<0.001 **
PN (kcal/day)			
Day1	80.6	144.0	0.042 *
Day2	122.8	140.1	0.629
Day3	135.3	182.8	0.257
Day4	105.5	224.3	0.007 **
Day5	127.5	278.3	0.001 **
Day6	128.5	242.4	0.019 *
Day7	123.6	290.2	0.001 **
EN/EN + PN (%)			
Day1	85.9	75.2	0.029 *
Day2	78.7	74.1	0.413
Day3	78.0	70.7	0.157
Day4	83.2	66.1	0.005 **
Day5	81.0	61.7	<0.001 **
Day6	82.8	67.0	0.006 **
Day7	84.9	60.9	<0.001 **

EN: enteral nutrition; PN: parenteral nutrition; * *p* < 0.05, ** *p* < 0.01.

**Table 3:** Adjusted hazard ratios of hospital mortality.

	Simple Model			Multiple Model		
	HR	(95% CI)	*p*-Value	HR	(95% CI)	*p*-Value
Age	1.02	(1.00–1.03)	0.040 *	1.01	(1.00–1.03)	0.126
Apache II	1.08	(1.03–1.12)	0.001 **	1.05	(1.00–1.10)	0.046 *
Sex (female vs. male)	1.19	(0.72–1.96)	0.502	1.43	(0.86–2.38)	0.166
Weight	0.99	(0.97–1.01)	0.268			
Body mass index	1.00	(0.95–1.06)	0.928			
Average 7 day						
EN/EN + PN (%)	0.97	(0.96–0.98)	<0.001 **	0.97	(0.96–0.99)	<0.001 **
SOFA	0.99	(0.92–1.07)	0.862			
**Comorbidity**						
Diabetes mellitus	1.13	(0.66–1.91)	0.663			
Hypertension	1.18	(0.71–1.96)	0.517			
Congestive heart failure	1.17	(0.47–2.94)	0.733			
Liver cirrhosis	1.14	(0.56–2.33)	0.716			
COPD	0.88	(0.42–1.86)	0.743			
Immunocompromised host	1.07	(0.62–1.85)	0.819			
Hemodialysis	0.84	(0.50–1.40)	0.501			
Sepsis	1.44	(0.87–2.40)	0.155			

APACHE II: Acute Physiology and Chronic Health Evaluation II; EN, enteral nutrition; PN, parenteral nutrition; SOFA, Sequential Organ Failure Assessment; COPD, chronic obstructive pulmonary disease; Cox regression, * *p* < 0.05, ** *p* < 0.01.
